# Evaluation of Deviation From Planned Cohort Size and Operating Characteristics of Phase 1 Trials

**DOI:** 10.1001/jamanetworkopen.2020.37563

**Published:** 2021-02-17

**Authors:** Minjeong Park, Suyu Liu, Timothy Anthony Yap, Ying Yuan

**Affiliations:** 1 Division of Biometrics 1, Office of Biostatistics, Office of Translational Sciences, Center for Drug Evaluation and Research Food and Drug Administration, Silver Spring, Maryland; 2 Department of Biostatistics, The University of Texas MD Anderson Cancer Center, Houston; 3 Department of Investigational Cancer Therapeutics, The University of Texas MD Anderson Cancer Center, Houston; 4 Khalifa Institute for Personalized Cancer Therapy, The University of Texas MD Anderson Cancer Center, Houston; 5 The Institute for Applied Cancer Science, The University of Texas MD Anderson Cancer Center, Houston

## Abstract

**Importance:**

The cohort size of phase 1 clinical trials and thus the timing of the interim decisions are typically prespecified in the trial protocol. During trial implementation, however, the cohort size often deviates from the planned one, which shifts the schedule of the interims. Despite its pervasiveness in phase 1 trials, the association of cohort size deviation with the operating characteristics of these trials is not clear.

**Objective:**

To explore the association between cohort size deviation and the operating characteristics of phase 1 clinical trials.

**Design, Setting, and Participants:**

In this cross-sectional simulation study, a review was conducted of 102 phase 1 dose-escalation trials published between January 2017 and May 2018 in 3 peer-reviewed journals (*Journal of Clinical Oncology*, *Clinical Cancer Research*, and *Cancer*). After exclusion of studies that did not report the cohort size, 45 trials remained for analysis. Based on the analysis results, a simulation study was performed to systematically investigate the association of cohort size deviation with the operating characteristics of the trials.

**Main Outcomes and Measures:**

The prevalence of cohort size deviation and the percentage of correct selection of the maximum tolerated dose.

**Results:**

Of the 45 reviewed trials, 10 (22.2%) adhered strictly to the planned cohort size. The simulation study showed that when cohort size deviation was random, it had little association with the performance of novel model-based and model-assisted designs (mean reduction in the percentage of correct selection of the maximum tolerated dose was 0.87 percentage point for the continual reassessment method and 0.84 percentage point for the bayesian optimal interval design). When the cohort size deviation was informative and made based on the observed data on toxicity (eg, if dose-limiting toxicity was observed, the size of the next or current cohort was reduced or expanded), the variation of the design performance increased. The range of the change in the percentage of correct selection was −3.7 to 1.3 percentage points for the continual reassessment method and −4.5 to 0 percentage points for the bayesian optimal interval design.

**Conclusions and Relevance:**

The findings suggest that when novel phase 1 clinical trial designs are used, some cohort size deviation is acceptable and has little association with the performance of the designs. These deviations may be used by expert investigators to properly interpret the data, ensure safety, and leverage flexibility in the protocol.

## Key Points

**Question:**

Is there an association between cohort size deviation and the operating characteristics of phase 1 trials?

**Findings:**

In this cross-sectional simulation study of 45 phase 1 studies, cohort size deviation had little association with the performance of novel model-based and model-assisted designs, including continuous reassessment method and bayesian optimal interval design.

**Meaning:**

The findings suggest that some cohort size deviation is acceptable and had little association with the performance of phase 1 trial designs.

## Teaser

This cross-sectional study evaluates the association of cohort size deviation with the operating characteristics in phase 1 clinical trials using simulation models.

## Introduction

The primary objective of phase 1 oncology trials, particularly for cytotoxic agents, is to establish the maximum tolerated dose (MTD) of a new drug.^1^ For novel targeted therapies and immunotherapies, the identification of the MTD may be less critical, but is still of practical interest and importance because the MTD determines the range of safe doses from which the recommended phase 2 dose or optimal biologic dose can be selected by accounting for other clinical outcomes (eg, pharmacokinetic and pharmacodynamic outcomes). To protect patients from toxic or subtherapeutic doses, investigators in phase 1 trials provide treatment in specific dosing cohorts (eg, 3 patients) in a “learn-as-we-go” fashion (ie, making dose escalation or deescalation decisions on the basis of accrued data after a cohort of patients completes their dose-limiting toxicity [DLT] assessment).

The size of the cohort is typically prespecified in the trial protocol (eg, 3 patients) to facilitate the trial implementation and evaluation of the operating characteristics of the design. The actual cohort size used when implementing the trial, however, often deviates from the prespecified cohort size. Consider a phase 1 trial with the planned cohort size of 3 patients and a 28-day DLT assessment window. There are various reasons that the cohort size may deviate. For example, with targeted agents and immunotherapy, chronic low-grade toxicity may not qualify as a formal DLT or high-grade toxicity may not be clearly attributable to the treatment. In these cases, an additional 1 to 3 patients may be enrolled to receive the current dose to obtain a better understanding of the toxicity profile, resulting in an overenrolled cohort with 4 or 6 patients. Underenrolling also occurs in practice. The most common reason for underenrollment is patient who are inevaluable or drop out of the trial. Suppose 3 patients in the current cohort are enrolled at days 1, 7, and 15 and at day 38, the third patient was found to be inevaluable; at that point, only DLT data from the first 2 patients would be available, resulting in an incomplete cohort with 2 patients. Ideally, the inevaluable patient should be replaced to complete the current cohort before enrolling the next cohort; however, that can substantially slow the progress of the trial because patient who was replaced will have to be followed up for another 28 days to evaluate toxicity before enrolling the next cohort. Owing to such logistics, investigators often prefer not to replace the inevaluable patient, favoring real-time decision-making for dose escalation or deescalation based on the available data. These cohort size deviations may affect the operating characteristics of the trial design because they change the timing of dose escalation and deescalation decisions. For example, given the planned cohort size of 3, without any deviations, the decision of dose escalation or deescalation would be made on the basis of the interim data from 3, 6, and 9 (evaluable) patients and so on. However, in the presence of deviations, the decisions would be made on the basis of fewer or more patients. Of note, replacing inevaluable patients (eg, who failed to receive the minimal required treatment) in a cohort is not regarded as a deviation here because it actually results in a complete cohort (eg, 3 evaluable patients).

Should these cohort size deviations be allowed? And how are they associated with the operating characteristics of the trial design? On the basis of our experiences serving on institutional research boards and data safety monitoring boards, cohort size deviation is one of the most common issues encountered and debated, and committees often have different views on the appropriateness of such adjustments. Some researchers believe that this might invalidate design operating characteristics presented in the protocol, which are based on a fixed, prespecified cohort size. Other researchers, however, assert that such deviations may be acceptable, providing clinicians a useful tool to handle complicated trial practice and logistic issues.

Studying the issue of cohort size deviation is of particular need given the increasing acceptance and use of novel phase 1 trial designs,^2,3^ such as the continuous reassessment method (CRM)^4^ and its various extensions^5,6,7,8,9^ (known as model-based designs) and the bayesian optimal interval (BOIN) design^10^ and the keyboard design^11^ (known as model-assisted designs). These novel designs statistically allow for decision-making regarding dose escalation or deescalation in the presence of cohort size deviation. However, it is unclear whether cohort size deviation invalidates their operating characteristics. The goal of this article was to systematically investigate this association of cohort size deviation with the operating characteristic of phase 1 trials and provide some practical guidance based on statistical rationale and evidence. To our knowledge, this article is the first study to systematically investigate this issue.

## Methods

In this cross-sectional simulation study, we reviewed 102 phase 1 dose-escalation trials published between January 2017 and May 2018, obtained by searching for the keywords *phase I*, *dose escalation*, and *dose finding* in 3 peer-reviewed journals (*Journal of Clinical Oncology*, *Clinical Cancer Research*, and *Cancer*). The primary objective of the review was to obtain an estimate of cohort size deviation to appropriately set up simulation parameters. Among the 102 trials, we excluded 57 (56%) that did not provide information on the cohort size actually used but only provided the aggregated data on the total number of patients treated at each dose. Thus, a total of 45 trials were used to summarize cohort size deviations. All of these trials had a planned cohort size of 3 except for 1 that had four. Only 10 trials (22%) complied with their initially planned cohort size. Because this was a simulation study that did not involve human participants, The University of Texas MD Anderson Cancer Center waived the need for institutional review board approval. This study followed the Strengthening the Reporting of Observational Studies in Epidemiology (STROBE) reporting guideline.

### Continual Reassessment Method

The CRM is a model-based design that assumes a parametric model for the dose-toxicity curve.^4^ The trial starts with treating the first cohort of patients at the lowest dose or prespecified starting dose. After each patient cohort is treated, the CRM updates the estimate of the dose-toxicity curve based on the accumulating DLT data and assigns the next cohort to the dose for which the estimate of the DLT probability is closest to the target DLT probability ϕ (eg, ϕ = 0.2 or 0.3). The trial continues in this manner until reaching the prespecified maximum sample size. At that point, the MTD is selected as the dose with an estimated DLT probability closest to ϕ. Because it is possible to update the estimate of the dose-toxicity curve at any time during the trial based on the actually observed data, the CRM has the capability to make a dose escalation or deescalation decision when the number of patients deviates from the planned cohort size.

### BOIN Design

The BOIN is a model-assisted design.^10,12^ Unlike the CRM, it does not assume a parametric model on the dose-toxicity curve. The BOIN design makes the decision of dose escalation or deescalation simply by comparing the observed DLT rate *p̂_cur_* at the current with prespecified dose escalation (*λ_e_*) and deescalation (*λ_d_*) boundaries, where *p̂_cur_* = (the number of patients who experienced DLT at the current dose)/(the number of patients treated at the current dose), as follows:

- If *p̂_cur_* ≤ *λ_e_*, escalate to the next higher dose.
- If *p̂_cur_* > *λ_d_*, de-escalate to the next lower dose.
- Otherwise, stay at the current dose.

eFigure 1 in the Supplement provides the values of *λ_e_* and *λ_d_* for some commonly used target DLT probability ϕ. For example, *λ_e_* = 0.157 and *λ_d_* = 0.238 when target ϕ = 0.2; and *λ_e_* = 0.157 and *λ_d_* = 0.238 when target ϕ = 0.3. The trial continues in this manner until reaching the prespecified maximum sample size. As the BOIN makes decisions based on the observed DLT rate at the current dose, it allows for a dose escalation or deescalation decision when the number of patients deviates from the planned cohort size. For example, suppose 4 patients rather than 3 patients are treated and 1 DLT is observed, the observed DLT rate is 1/4, which can be compared *λ_e_* and *λ_d_* to make the decision of dose escalation or deescalation. More details are provided in the eMethods and eFigure 1 in the Supplement).

### Statistical Analysis

**Figure 1.**
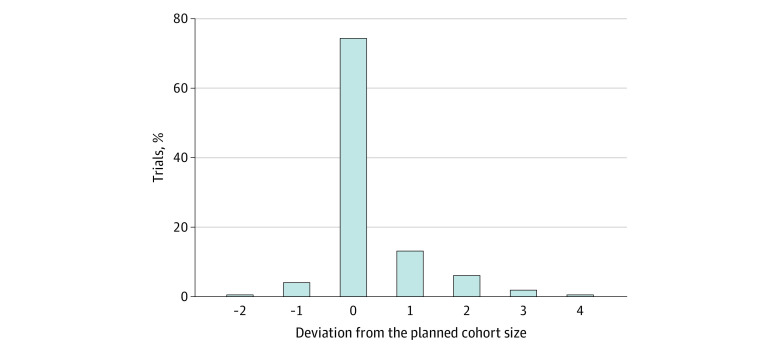
Cohort Size Deviation in Phase 1 Clinical Trials Zero indicates that the actual cohort size used was consistent with the planned cohort size.

For the Monte Carlo experiment, computer simulations were performed to evaluate the operating characteristics of the 3 + 3, CRM, and BOIN designs when the cohort sizes deviated from the planned size. We considered 5 dose levels with a maximum sample size of 30. The planned cohort size was 3 for consistency with most trials in practice. We considered 2 mechanisms of cohort size deviation: random and informative. Under the random mechanism, the size of each cohort was generated randomly from its empirical distribution observed in the real trials (Figure 1) independent of the interim data on toxicity. We considered 3 informative mechanisms in which the cohort size deviation depended on the interim data on toxicity: (1) if any DLT was observed in the current cohort, we expanded the size of the next cohort to 4; (2) if any DLT was observed in the current cohort, we reduced the size of the next cohort to 2; and (3) if any DLT was observed in the current cohort, we expanded the size of the current cohort to 4. We considered the target DLT probability ϕ = 20% or 30%. For each target DLT rate, we considered 9 scenarios for toxicity, representing a variety of dose-toxicity associations (eTable 1 in the Supplement). Under each scenario, we simulated 5000 trials. For the 3 + 3 design, we only present the results for the case in which the planned cohort size was strictly followed because the 3 + 3 design does not define the rule for dose escalation or deescalation when the number of patients deviates from 3 and 6. In addition, because the 3 + 3 design often stopped the trial early (eg, when 2 of 3 patients experienced DLTs) before reaching 30 patients, in these cases, the remaining patients were treated at the selected MTD as the cohort expansion such that the 3 designs had comparable sample sizes. The configurations for the CRM and the BOIN are provided in the eMethods in the Supplement). We considered the following 3 metrics to measure the performance of the designs, calculated based on 5000 simulated trials: the percentage of correct selection (PCS) of the MTD, the mean number of patients allocated to the MTD, and the mean number of patients allocated to the doses above the MTD. Analyses were performed using R, version 3.2.3 (R Project for Statistical Computing).

## Results

### Cohort Size Deviation in Practice

Figure 1 shows the distribution of the cohort size deviation in 45 trials, with the value of 0 indicating compliance with the planned cohort size. Deviations ranged from −3 (ie, 3 patients less than planned) to 4 (ie, 4 patients more than planned). A mean of 85 of 331 cohorts (25.7%) in the 45 trials deviated from the planned size, and the number of deviations was 1.9 per trial. None of the trials provided details on how or why these cohort size deviations occurred or how they were handled. A total of 42 (93%) trials used the 3 + 3 design and its variations (eg, accelerated titration), which typically cannot handle a cohort size deviation from 3 and 6 except when 2 or more DLTs are observed at a dose. The dose levels investigated in these studies ranged from 3 to 9 with a median of 5.5 doses (interquartile range, 4-7 doses). The median total sample size was 67 individuals (range, 9-67 individuals). Therefore, in our simulation study, we chose 5 doses and the maximum sample size of 30 (ie, the maximum sample size of the 3 + 3 design with 5 doses).

### Random Cohort Size Deviation

**Figure 2.**
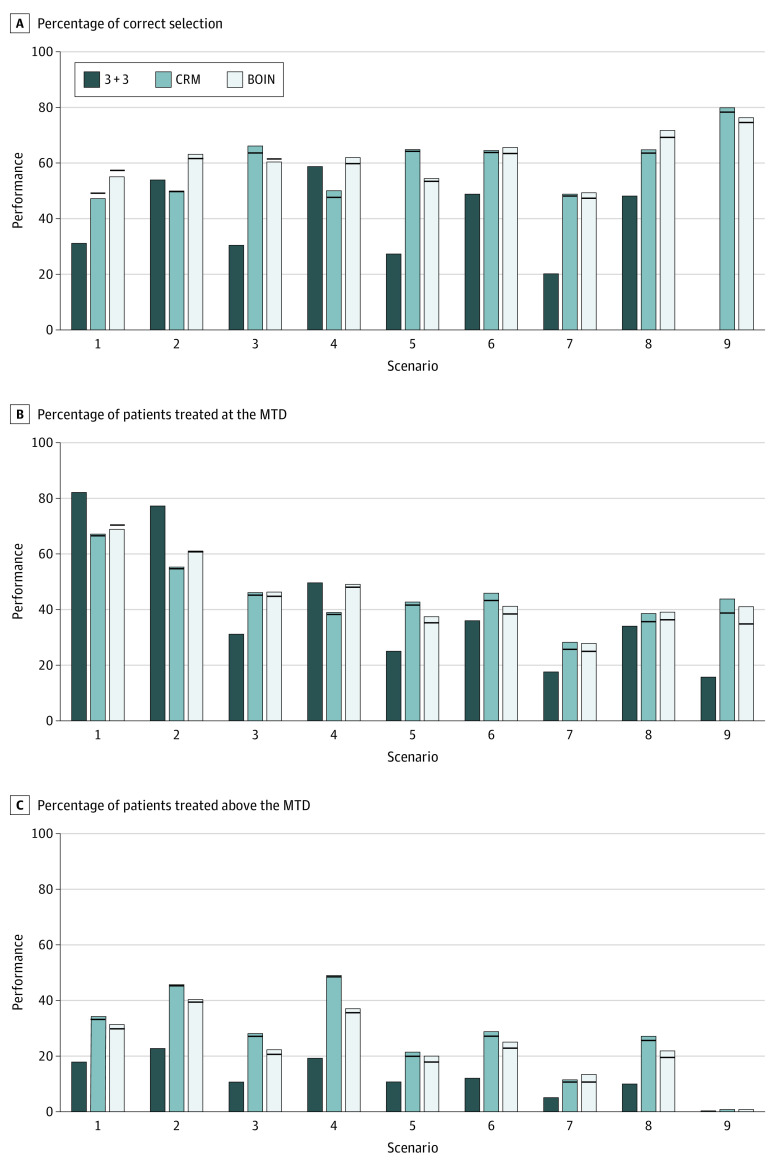
Simulation Results of the 3 + 3, Continual Reassessment Method (CRM), and Bayesian Optimal Interval (BOIN) Designs When the Cohort Deviation Was Generated According to the Frequency Observed in Real Trials The bars show the performance (percentage) of the designs when the planned cohort size was strictly followed; the horizonal lines indicate the value of the performance metric in the presence of cohort size deviation. The target dose-limiting toxicity rate was 0.3. MTD indicates maximum tolerated dose.

Figure 2 shows the results when cohort size deviation followed what was observed in real trials when the target DLT probability was 30%. The CRM and the BOIN yielded substantially higher accuracy in identifying the MTD than the 3 + 3 design. The mean PCS of the MTD for the CRM and the BOIN was 25 percentage point and 26 percentage points, respectively, higher than that of the 3 + 3 design (35%). In addition, the CRM and the BOIN allocated more patients to the MTD (difference, 4-5 percentage points, respectively). The 3 + 3 design was the most conservative, assigning fewer patients to doses above the MTD than the CRM (difference, 15 percentage points) and fewer patients to the doses above the MTD than the BOIN (difference, 11 percentage points).

Cohort size deviation showed little association with the performance of the CRM and the BOIN. Compared with the counterpart that strictly followed the planned cohort size of 3, in the presence of cohort size deviation, the mean PCS of CRM was reduced by 0.87 percentage points (range, −2.5 to 1.96 percentage points), and the mean PCS of the BOIN was reduced by 0.84 percentage points (range, −2.3 to 2.5 percentage points) across 9 dose-toxicity scenarios. The percentage of patients treated above the MTD was reduced by a mean of 0.75 percentage points (range, −1.6 to 0.25 percentage points) for the CRM and by a mean of 1.5 percentage points (range, 0-2.5 percentage points) for the BOIN. The results for the target DLT rate of 20% were similar and are provided in eFigure 2 in the Supplement; the detailed results with the selection percentage and the number of patients allocated to receive each dose are provided in eTables 2 and 3 in the Supplement.

The distribution of the cohort size deviation used in our simulation was estimated based on 45 trials, with the other 57 trials excluded owing to the lack of cohort-by-cohort dose assignment information. One potential concern is the generalizability of our findings. To address this, we conducted a sensitivity analysis by increasing the percentage of cohorts deviated from the planned size from 25.7% (observed in the trials) to 50%. The results (eFigures 3 and 4 in the Supplement) showed that our findings were robust and consistent with the above assertions; specifically, the deviation showed little association with the operating characteristics of the design.

### Informative Cohort Size Deviation

**Figure 3.**
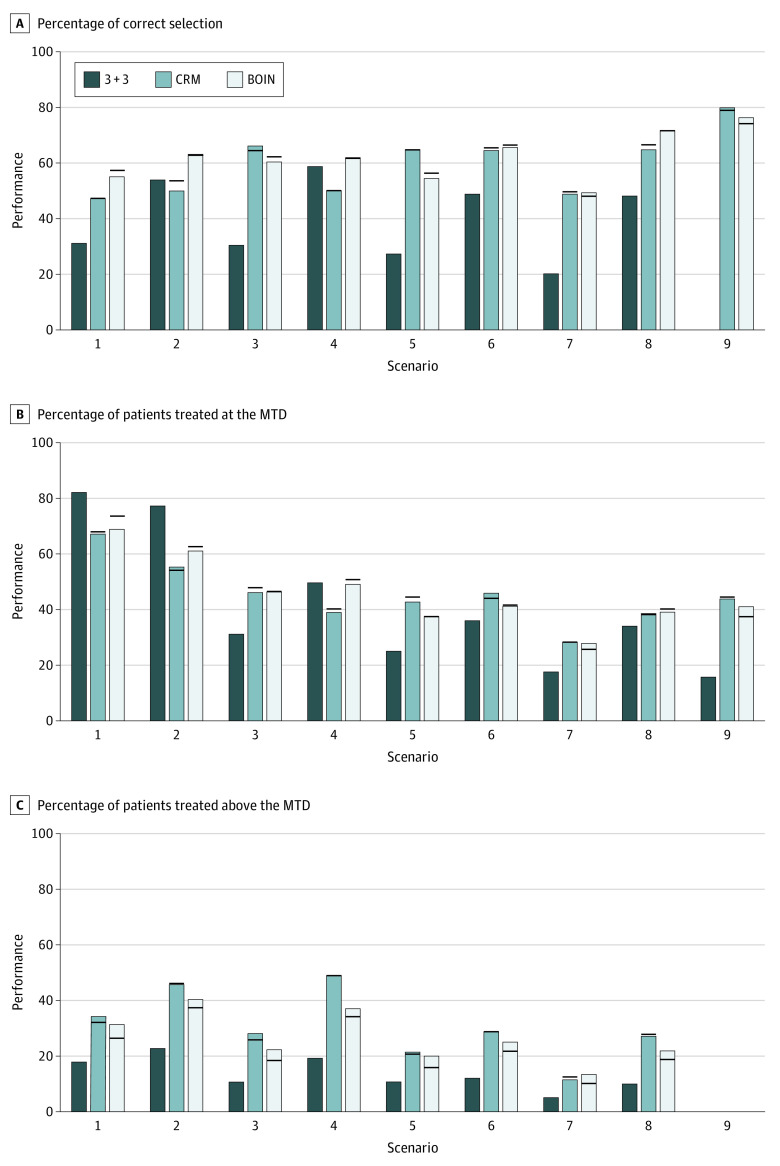
Simulation Results of the 3 + 3, Continual Reassessment Method (CRM), and Bayesian Optimal Interval (BOIN) Designs Under Informative Cohort Size Deviation With Expansion of the Next Cohort Size If any dose-limiting toxicity (DLT) was observed in the current cohort, the size of the next cohort was expanded to 4. The bars show the performance (percentage) of the designs when the planned cohort size was strictly followed; horizonal lines indicate the value of the performance metric in the presence of cohort size deviation. The target DLT rate was 0.3. MTD indicates maximum tolerated dose.

**Figure 4.**
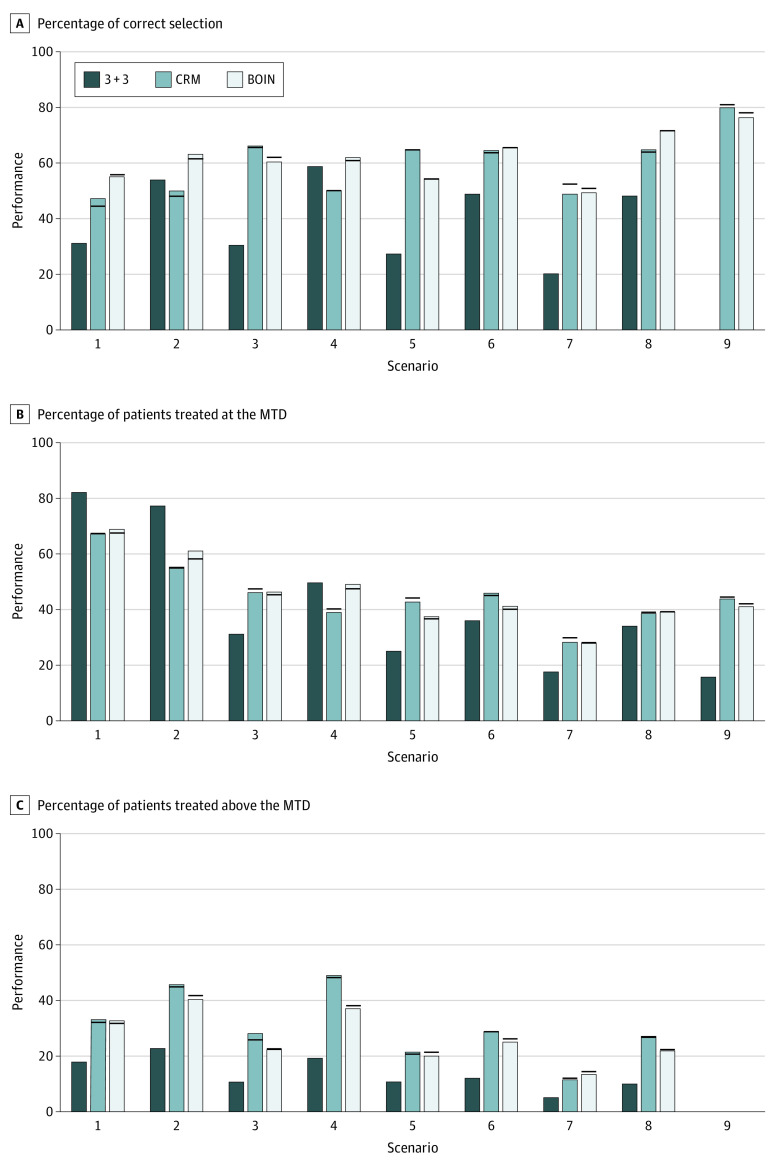
Simulation Results of the 3 + 3, Continual Reassessment Method (CRM), and Bayesian Optimal Interval (BOIN) Designs Under Informative Cohort Size Deviation With Reduction of the Next Cohort Size If any dose-limiting toxicity (DLT) was observed in the current cohort, the size of the next cohort was reduced to 2. The bars show the performance (percentage) of the designs when the planned cohort size was strictly followed; horizonal lines indicate the value of the performance metric in the presence of cohort size deviation. The target DLT rate was 0.3. MTD indicates maximum tolerated dose.

**Figure 5.**
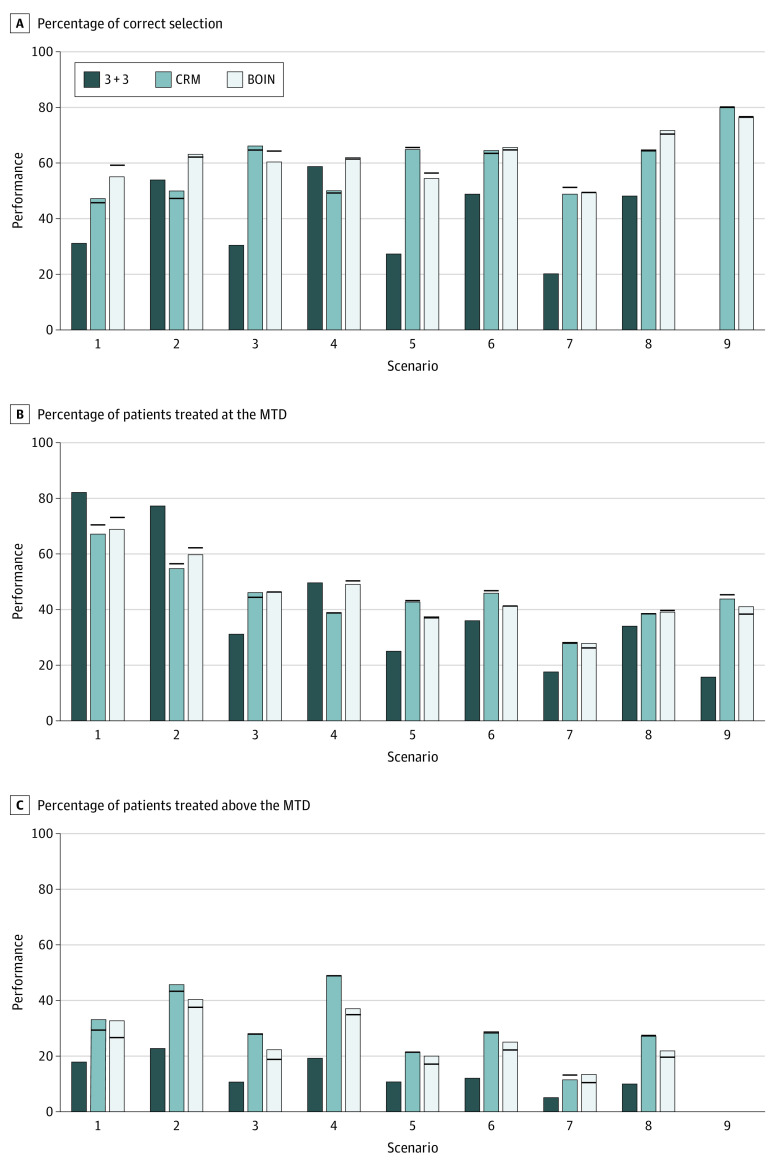
Simulation Results of the 3 + 3, Continual Reassessment Method (CRM), and Bayesian Optimal Interval (BOIN) Designs Under Informative Cohort Size Deviation With Expansion of the Present Cohort Size If any dose-limiting toxicity (DLT) was observed in the current cohort, the size of the current cohort was expanded to 4. The bars show the performance (percentage) of the designs when the planned cohort size was strictly followed; horizonal lines indicate the value of the performance metric in the presence of cohort size deviation. The target DLT rate was 0.3. MTD indicates maximum tolerated dose.

Figure 3, Figure 4, and Figure 5 show the results for the target DLT probability of 0.3 under 3 different types of informative cohort size deviation. Overall, the CRM and the BOIN were robust to the informative cohort size deviation. The accuracy of selecting the MTD was not affected, and the mean change in PCS was less than 0.5 percentage points compared with the counterpart that strictly followed the planned cohort size of 3. Cohort size deviation had an association with the percentage of patients treated at the MTD and the percentage of patients treated above the MTD. The range of change in these 2 metrics was wider than that under the random cohort size deviation but minor. For example, with a third type of informative cohort size deviation, the mean change in the percentage of patients treated above the MTD was −0.4 percentage points (range, −3.7 to 1.3 percentage points) for the CRM and −2.4 percentage points (range, −4.5 to 0 percentage points) for the BOIN. The results for a target DLT probability of 20% were similar (eFigure 5-7 in the Supplement).

## Discussion

Cohort size deviation is one of the most common issues in phase 1 trials, with to our knowledge, no guidelines or studies undertaken to systematically evaluate the association of cohort size deviation with the operating characteristics of phase 1 trials. We demonstrated that cohort size deviation had little association with the performance of novel, model-based and model-assisted designs, including the CRM and the BOIN, in particular when the cohort size deviation was caused by external reasons that are not related to the observed data on toxicity. When the cohort size deviation was informative and made based on the observed data on toxicity (eg, if a DLT was observed, the size of the next or current cohort is reduced or expanded), the variation of the design performance was increased slightly. Our results support the adoption of novel trial designs (eg, the BOIN and the CRM), in addition to high efficiency for identifying the MTD,^2^ to replace the 3 + 3 design. The ability of the BOIN and the CRM to make real-time decisions in the presence of overenrolled or underenrolled cohorts may increase the flexibility and reduce the logistical difficulties for implementing trials. In contrast, the 3 + 3 design does not have any built-in rules to handle most cohort size deviations (ie, no rule is defined when the number of patients deviates from 3 and 6), and investigators have to resort to additional, often subjective rules to make decisions of dose escalation or deescalation that typically are not described in the trial protocol or publication. In addition, our simulation showed that the 3 + 3 design was overly conservative, consistent with previous research.^8,9,10^ Although safety is desirable, being overly conservative is undesirable, resulting in poor accuracy for identifying the MTD. Because the dose selected in phase 1 trials is used in subsequent phase 2 trials to treat a larger number of patients, misidentification of the MTD may lead to the treatment of a large number of patients at overly toxic or subtherapeutic doses. The CRM and BOIN designs generally have a better balance in safety (ie, risk of overdosing) and identifying the MTD.^8,10^

### Limitations

This study has limitations. The literature review performed for studying the cohort deviation was limited to 3 cancer-related journals, and 56% of the studies were excluded owing to the lack of cohort-by-cohort dose assignment information. As a result, the distribution of the cohort deviation observed in these studies may not be fully generalizable and subject to potential selection and publication bias. This may not be a major concern here, however, because the primary objective of this study was not to accurately characterize the cohort deviation but to evaluate its association with the operating characteristics of the trial designs. The observed distribution of the cohort deviation was used as one of the simulation settings, and our simulation study also considered other mechanisms of cohort deviations as well as more extreme cases as the sensitivity analysis. The simulation results supported the robustness of our findings and conclusions.

## Conclusions

The findings of this cross-sectional simulation study suggest that when using novel, phase 1 trial designs, such as the CRM and the BOIN, some cohort size deviation may be generally acceptable and has little association with the performance of the designs. These deviations may be useful for expert investigators to properly interpret data, ensure safety, and leverage flexibility in the protocol, providing a motivation to use the novel designs to replace the 3 + 3 design.

## Notes

Supplement. **eMethods.** Simulation Setup for Continual Reassessment Method (CRM) and Bayesian Optimal Interval (BOIN) Designs **eFigure 1.** The Flowchart of the Bayesian Optimal Interval (BOIN) Design **eTable 1.** Eighteen Dose-Toxicity Scenarios Considered in the Simulation Study **eTable 2.** Simulation Results Under 9 Dose-Toxicity Scenarios With the Target DLT Probability of 0.3 **eTable 3.** Simulation Results Under 9 Dose-Toxicity Scenarios With the Target DLT Probability of 0.2 **eFigure 2.** Simulation Results of the 3+3, Continual Reassessment Method (CRM), and Bayesian Optimal Interval (BOIN) Designs When the Cohort Deviation was Generated According to the Frequency Observed in Real Trials, and the Target DLT Rate Was 0.2 **eFigure 3.** Sensitivity Analysis When the Percentage of Cohort Deviation Was Increased From 25.7% (the Rate Observed in the Literature Review) to 50%, With the Target DLT Rate of 0.3 **eFigure 4.** Sensitivity Analysis When the Percentage of Cohort Deviation Was Increased From 25.7% (the Rate Observed in the Literature Review) to 50%, With the Target DLT Rate of 0.2 **eFigure 5.** Simulation Results of the 3+3, Continual Reassessment Method (CRM), and Bayesian Optimal Interval (BOIN) Designs Under Informative Cohort Size Deviation With Expansion of the Next Cohort Size, and the Target DLT Rate of 0.2 **eFigure 6.** Simulation Results of the 3+3, continual reassessment method (CRM), and Bayesian Optimal Interval (BOIN) Designs Under Informative Cohort Size Deviation With Reduction of the Next Cohort Size, and the Target DLT Rate of 0.2 **eFigure 7.** Simulation Results of the 3+3, Continual Reassessment Method (CRM), and Bayesian Optimal Interval (BOIN) Designs Under Informative Cohort Size Deviation With Expansion of the Present Cohort Size, and the Target DLT Rate of 0.2
