# Supplementary material for: Evaluation of Deviation From Planned Cohort Size and Operating Characteristics of Phase 1 Trials
Source: JAMA Netw Open. 2021 Feb 17;4(2):e2037563. doi: 10.1001/jamanetworkopen.2020.37563 (PMC7890531; doi:10.1001/jamanetworkopen.2020.37563)
Supplement: Supplement. — eMethods. Simulation Setup for Continual Reassessment Method (CRM) and Bayesian Optimal Interval (BOIN) Designs eFigure 1. The Flowchart of the Bayesian Optimal Interval (BOIN) Design eTable 1. Eighteen Dose-Toxicity Scenarios Considered in the Simulation Study eTable 2. Simulation Results Under 9 Dose-Toxicity Scenarios With the Target DLT Probability of 0.3 eTable 3. Simulation Results Under 9 Dose-Toxicity Scenarios With the Target DLT Probability of 0.2 eFigure 2. Simulation Results of the 3+3, Continual Reassessment Method (CRM), and Bayesian Optimal Interval (BOIN) Designs When the Cohort Deviation was Generated According to the Frequency Observed in Real Trials, and the Target DLT Rate Was 0.2 eFigure 3. Sensitivity Analysis When the Percentage of Cohort Deviation Was Increased From 25.7% (the Rate Observed in the Literature Review) to 50%, With the Target DLT Rate of 0.3 eFigure 4. Sensitivity Analysis When the Percentage of Cohort Deviation Was Increased From 25.7% (the Rate Observed in the Literature Review) to 50%, With the Target DLT Rate of 0.2 eFigure 5. Simulation Results of the 3+3, Continual Reassessment Method (CRM), and Bayesian Optimal Interval (BOIN) Designs Under Informative Cohort Size Deviation With Expansion of the Next Cohort Size, and the Target DLT Rate of 0.2 eFigure 6. Simulation Results of the 3+3, continual reassessment method (CRM), and Bayesian Optimal Interval (BOIN) Designs Under Informative Cohort Size Deviation With Reduction of the Next Cohort Size, and the Target DLT Rate of 0.2 eFigure 7. Simulation Results of the 3+3, Continual Reassessment Method (CRM), and Bayesian Optimal Interval (BOIN) Designs Under Informative Cohort Size Deviation With Expansion of the Present Cohort Size, and the Target DLT Rate of 0.2 [file jamanetwopen-e2037563-s001.pdf]

Supplemental Online Content

Park M, Liu S, Yap TA, Yuan Y. Evaluation of deviation from planned cohort size and operating characteristics of phase 1 trials. *JAMA Netw Open.* 2021;4(2):e2037563. doi:10.1001/jamanetworkopen.2020.37563

eMethods. Simulation Setup for Continual Reassessment Method (CRM) and Bayesian Optimal Interval (BOIN) Designs

eFigure 1. The Flowchart of the Bayesian Optimal Interval (BOIN) Design

eTable 1. Eighteen Dose-Toxicity Scenarios Considered in the Simulation Study

eTable 2. Simulation Results Under 9 Dose-Toxicity Scenarios With the Target DLT Probability of 0.3

eTable 3. Simulation Results Under 9 Dose-Toxicity Scenarios With the Target DLT Probability of 0.2

eFigure 2. Simulation Results of the 3+3, Continual Reassessment Method (CRM), and Bayesian Optimal Interval (BOIN) Designs When the Cohort Deviation was Generated According to the Frequency Observed in Real Trials, and the Target DLT Rate Was 0.2

eFigure 3. Sensitivity Analysis When the Percentage of Cohort Deviation Was Increased From 25.7% (the Rate Observed in the Literature Review) to 50%, With the Target DLT Rate of 0.3

eFigure 4. Sensitivity Analysis When the Percentage of Cohort Deviation Was Increased From 25.7% (the Rate Observed in the Literature Review) to 50%, With the Target DLT Rate of 0.2

eFigure 5. Simulation Results of the 3+3, Continual Reassessment Method (CRM), and Bayesian Optimal Interval (BOIN) Designs Under Informative Cohort Size Deviation With Expansion of the Next Cohort Size, and the Target DLT Rate of 0.2

eFigure 6. Simulation Results of the 3+3, continual reassessment method (CRM), and Bayesian Optimal Interval (BOIN) Designs Under Informative Cohort Size Deviation With Reduction of the Next Cohort Size, and the Target DLT Rate of 0.2

eFigure 7. Simulation Results of the 3+3, Continual Reassessment Method (CRM), and Bayesian Optimal Interval (BOIN) Designs Under Informative Cohort Size Deviation With Expansion of the Present Cohort Size, and the Target DLT Rate of 0.2

This supplemental material has been provided by the authors to give readers additional information about their work.

© 2021 Park M et al. *JAMA Network Open.*

eMethods

Simulation setup for continual reassessment method (CRM) and bayesian optimal interval (BOIN) designs

i) CRM
In CRM, we used the one-parameter power model, given by

$$p_j = a_j^{\exp(\alpha)}, \text{ for } j = 1, \dots, J,$$

where α is the unknown parameter, p_j is the DLT rate at dose j, and $0 < a_1 < \cdots < a_J < 1$ are prior guesses for the DLT probability at each dose, which are often called the "skeleton" of CRM. In our simulation, the prior estimates of DLT probability for five doses are (0.05, 0.1, 0.2, 0.3, 0.4) when target DLT probability ϕ=0.2, and are (0.08, 0.15, 0.3, 0.45, 0.55) when target DLT probability ϕ=0.3. We used normal prior α ~ N(0, 2).

The CRM starts the trial by treating the first cohort of patients at the lowest dose d_1. After each patient cohort is treated, the CRM updates the estimate of the dose-toxicity curve based on the accumulating DLT data across all dose levels, and it assigns the next cohort of patients to the dose whose posterior mean estimate of the DLT probability is closest to the target ϕ. In our simulation, we did not allow dose skipping, and we restricted the dose escalation/de-escalation to one level at a time.

With CRM, we imposed the following safety stopping rule: stop the trial if the posterior probability that the DLT probability of the lowest dose is greater than the target ϕ exceeds 0.95. That is, the trial will be terminated if

$$\Pr(p_1 > \phi | data) > 0.95.$$

© 2021 Park M et al. *JAMA Network Open.*

ii) BOIN

Let $\hat{p}_{cur}$ denote the observed DLT rate at the current dose, defined as $\hat{p}_{cur}$ = (the number of patients who experienced DLT at the current dose)/(the number of patients treated at the current dose). As shown in Figure S1, the BOIN design makes the decision of dose escalation/de-escalation simply by comparing the observed DLT rate $\hat{p}_{cur}$ with the following prespecified dose escalation (λ_e) and de-escalation (λ_d) boundaries:

- If $\hat{p}_{cur} \leq \lambda_e$, escalate to the next higher dose.
- If $\hat{p}_{cur} > \lambda_d$, de-escalate to the next lower dose.
- Otherwise, stay at the current dose.

In our simulation, we used default dose escalation and de-escalation boundaries provided by BOIN. That is, $\lambda_e = 0.157$ and $\lambda_d = 0.238$ when target ϕ = 0.2; and $\lambda_e = 0.157$ and $\lambda_d = 0.238$ when target ϕ = 0.3. The trial continues in this manner until reaching the prespecified maximum sample size. For patient safety, the BOIN design imposes an overdose control/early stopping rule as follows: if Pr($p_{cur} > \phi | data$) > 0.95 and at least 3 patients have been treated, the current and higher doses are eliminated from the trial. The trial is terminated if the lowest dose is eliminated. Pr($p_{cur} > \phi | data$) is calculated based on a beta-binomial model with a uniform prior on p_{cur}, i.e., $y|n \sim Binomial(p_{cur})$ and $p_{cur} \sim Unif(0, 1)$, where y is the number of patients with DLT at the current dose, and n is the total number of patients treated at the current dose.

© 2021 Park M et al. *JAMA Network Open.*

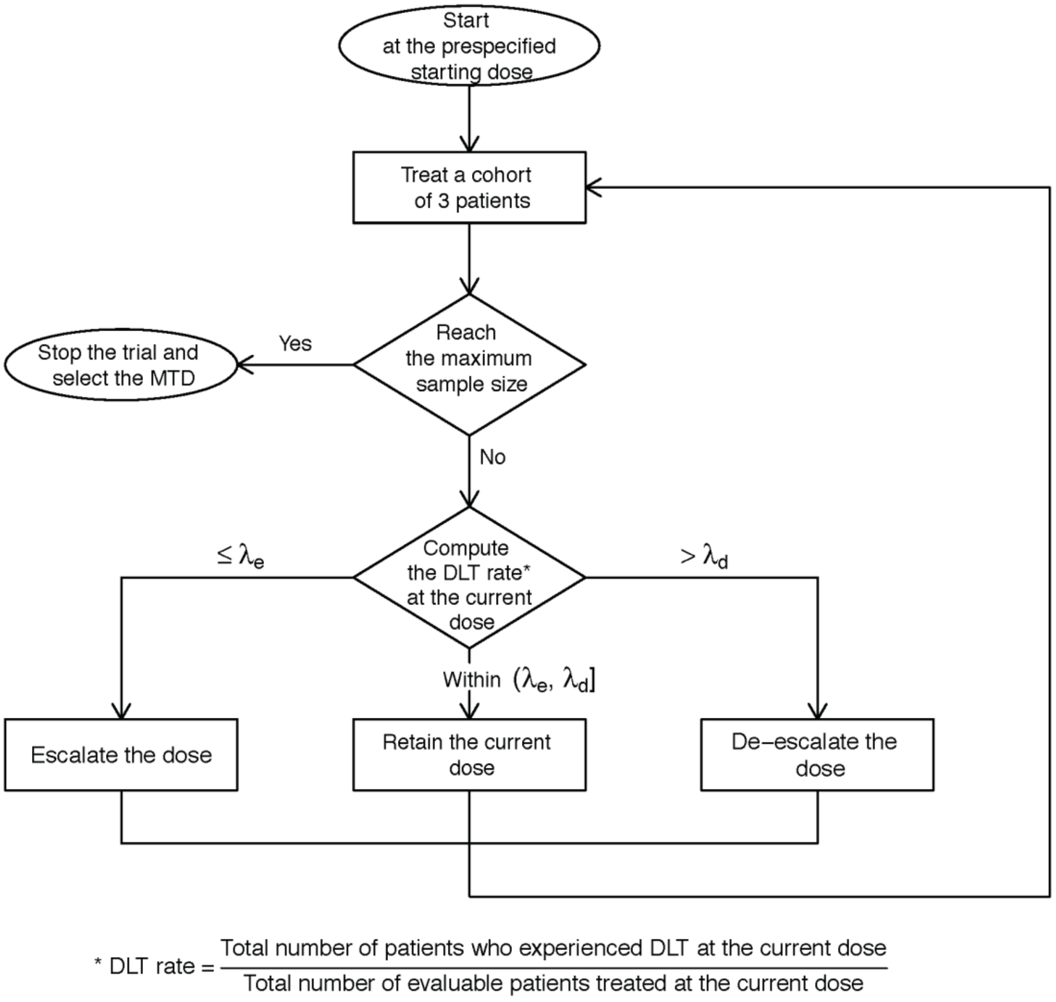

	Target toxicity rate ϕ					
Boundaries	0.15	0.20	0.25	0.30	0.35	0.40
λ_e (escalation)	0.118	0.157	0.197	0.236	0.276	0.316
λ_d (de-escalation)	0.179	0.238	0.298	0.358	0.419	0.479

eFigure 1. The flowchart of the bayesian optimal interval (BOIN) design, where λ_e and λ_d are the dose escalation boundary and de-escalation boundary, respectively. For the trial example with the target DLT rate of 0.3, $\lambda_e = 0.236$ and $\lambda_d = 0.358$.

eTable 1. Eighteen dose-toxicity scenarios considered in the simulation study. The doses in bold are the MTD. To reflect what may happen in practice, the MTD does not necessarily have the DLT probability equal to the target DLT probability.

Scenario	Dose level				
	1	2	3	4	5
**Target DLT probability = 0.3**					
1	**0.3**	0.4	0.5	0.6	0.7
2	**0.2**	0.44	0.55	0.6	0.7
3	0.12	**0.3**	0.45	0.6	0.7
4	0.01	**0.18**	0.45	0.6	0.8
5	0.08	0.12	**0.3**	0.42	0.55
6	0.01	0.14	**0.2**	0.5	0.58
7	0.07	0.12	0.17	**0.3**	0.44
8	0.01	0.02	0.07	**0.22**	0.5
9	0.02	0.04	0.06	0.12	**0.3**
**Target DLT probability = 0.2**					
1	**0.2**	0.35	0.4	0.5	0.6
2	**0.14**	0.32	0.35	0.45	0.55
3	0.1	**0.2**	0.35	0.45	0.5
4	0.01	**0.08**	0.35	0.45	0.55
5	0.04	0.06	**0.2**	0.35	0.48
6	0.01	0.07	**0.12**	0.37	0.5
7	0.05	0.06	0.09	**0.2**	0.37
8	0.01	0.02	0.04	**0.08**	0.38
9	0.05	0.06	0.07	0.08	**0.2**

© 2021 Park M et al. *JAMA Network Open.*

eTable 2. Simulation results under 9 dose-toxicity scenarios with the target DLT probability of 0.3. The doses in bold are the MTD. To reflect what may happen in practice, the MTD does not necessarily have the DLT probability equal to the target DLT probability. CRM-nd and BOIN-nd refer to CRM and BOIN with no cohort deviations.

Method	Scenario	Dose Level				
		1	2	3	4	5
	1	**0.3**	0.4	0.5	0.6	0.7
3+3	Selection %	31.7	10.2	1.8	0.1	0.0
	No. of patients	11.2	4.1	1.0	0.1	0.0
CRM-nd	Selection %	47.3	26.3	4.1	0.2	0.0
	No. of patients	15.5	7.2	2.3	0.2	0.0
CRM	Selection %	49.2	25.7	4.3	0.2	0.0
	No. of patients	15.9	7.1	2.2	0.2	0.0
BOIN-nd	Selection %	55.1	22.9	4.5	0.5	0.0
	No. of patients	16.9	6.8	1.9	0.3	0.0
BOIN	Selection %	57.6	22.4	3.7	0.2	0.0
	No. of patients	17.5	6.8	1.5	0.2	0.0
	2	**0.2**	0.44	0.55	0.6	0.7
3+3	Selection %	53.6	11.9	1.1	0.1	0.0
	No. of patients	15.6	5.4	1.0	0.1	0.0
CRM-nd	Selection %	50.5	41.6	2.8	0.1	0.0
	No. of patients	14.8	10.7	2.3	0.2	0.0
CRM	Selection %	49.8	42.3	3.0	0.2	0.0
	No. of patients	15.0	10.5	2.2	0.2	0.0
BOIN-nd	Selection %	60.7	33.1	2.9	0.3	0.0
	No. of patients	16.6	9.7	1.9	0.2	0.0
BOIN	Selection %	61.7	32.7	2.6	0.1	0.0
	No. of patients	16.8	9.7	1.5	0.1	0.0
	3	0.12	**0.3**	0.45	0.6	0.7
3+3	Selection %	48.0	30.4	6.5	0.3	0.0
	No. of patients	13.9	9.2	3.0	0.5	0.0
CRM-nd	Selection %	12.2	62.8	23.5	0.7	0.0
	No. of patients	7.4	13.5	6.9	0.9	0.1
CRM	Selection %	11.9	63.7	23.0	0.9	0.0
	No. of patients	7.8	13.1	6.8	0.9	0.1
BOIN-nd	Selection %	19.2	60.3	18.6	1.5	0.1
	No. of patients	9.6	12.9	5.4	0.9	0.1
BOIN	Selection %	18.0	61.6	18.4	1.5	0.1
	No. of patients	9.7	13.0	5.2	0.8	0.0
	4	0.01	**0.18**	0.45	0.6	0.8
3+3	Selection %	29.4	57.8	11.9	0.6	0.0
	No. of patients	9.4	14.5	5.2	0.8	0.1
CRM-nd	Selection %	0.5	48.5	49.7	1.4	0.0
	No. of patients	3.4	11.4	12.3	1.8	0.2
CRM	Selection %	0.3	47.7	50.2	1.8	0.0
	No. of patients	3.9	11.0	11.9	1.8	0.1
BOIN-nd	Selection %	2.1	59.8	36.1	2.0	0.1
	No. of patients	4.3	14.0	9.1	1.5	0.1

© 2021 Park M et al. *JAMA Network Open.*

BOIN	Selection %	1.9	59.8	36.0	2.2	0.0
	No. of patients	4.7	13.9	8.9	1.3	0.1
	5	0.08	0.12	**0.3**	0.42	0.55
3+3	Selection %	14.0	44.5	25.9	6.8	0.0
	No. of patients	6.7	11.2	7.2	2.6	0.4
CRM-nd	Selection %	0.2	12.2	64.0	21.4	2.1
	No. of patients	3.9	6.4	12.6	5.0	1.1
CRM	Selection %	0.1	13.2	64.2	20.5	1.9
	No. of patients	4.5	6.6	12.0	4.8	0.9
BOIN-nd	Selection %	0.7	19.7	54.5	21.8	3.2
	No. of patients	4.4	8.3	10.4	4.7	1.1
BOIN	Selection %	0.7	20.2	53.9	22.3	2.7
	No. of patients	4.8	8.5	10.2	4.3	0.9
	6	0.01	0.14	**0.2**	0.5	0.58
3+3	Selection %	17.9	26.9	48.2	5.4	0.0
	No. of patients	7.1	8.7	10.3	3.3	0.4
CRM-nd	Selection %	0.0	3.7	63.7	31.4	1.2
	No. of patients	3.1	4.6	13.1	7.2	1.1
CRM	Selection %	0.0	3.7	63.8	31.2	1.2
	No. of patients	3.5	4.9	12.5	6.8	1.0
BOIN-nd	Selection %	0.7	8.6	65.4	23.3	2.0
	No. of patients	3.7	6.5	11.7	6.2	0.9
BOIN	Selection %	0.8	9.0	63.6	24.9	1.7
	No. of patients	4.0	7.0	11.1	6.0	0.7
	7	0.07	0.12	0.17	**0.3**	0.44
3+3	Selection %	12.4	20.5	33.1	20.0	0.0
	No. of patients	6.4	7.3	7.9	5.0	1.4
CRM-nd	Selection %	0.1	3.5	30.7	49.7	15.9
	No. of patients	3.7	4.8	8.8	8.0	3.7
CRM	Selection %	0.0	4.0	32.8	48.2	15.0
	No. of patients	4.3	5.1	8.9	7.4	3.1
BOIN-nd	Selection %	0.7	5.1	27.8	47.2	19.1
	No. of patients	4.2	5.6	7.8	7.7	3.7
BOIN	Selection %	0.5	5.8	27.7	47.4	18.5
	No. of patients	4.6	6.0	7.9	7.2	3.1
	8	0.01	0.02	0.07	**0.22**	0.5
3+3	Selection %	0.5	5.6	35.6	47.3	0.0
	No. of patients	3.5	4.4	8.6	9.6	2.8
CRM-nd	Selection %	0.0	0.0	3.0	64.2	32.8
	No. of patients	2.9	2.9	4.2	11.2	7.8
CRM	Selection %	0.0	0.0	2.8	63.6	33.6
	No. of patients	3.4	3.3	4.5	10.3	7.3
BOIN-nd	Selection %	0.0	0.1	6.1	70.5	23.3
	No. of patients	2.9	3.2	5.5	11.3	6.2
BOIN	Selection %	0.0	0.1	6.0	69.3	24.7
	No. of patients	3.4	3.6	5.7	10.5	5.6
	9	0.02	0.04	0.06	0.12	**0.3**
3+3	Selection %	2.0	4.0	12.8	39.4	0.0

© 2021 Park M et al. *JAMA Network Open.*

	No. of patients	3.9	4.3	5.4	8.0	3.8
CRM-nd	Selection %	0.0	0.0	1.0	19.1	79.8
	No. of patients	3.0	3.0	3.9	6.2	12.9
CRM	Selection %	0.0	0.0	0.9	20.7	78.3
	No. of patients	3.5	3.4	4.4	6.3	11.2
BOIN-nd	Selection %	0.0	0.2	1.0	23.1	75.7
	No. of patients	3.1	3.3	3.9	7.2	11.5
BOIN	Selection %	0.0	0.0	1.1	24.3	74.6
	No. of patients	3.5	3.8	4.3	7.1	10.1

© 2021 Park M et al. *JAMA Network Open.*

eTable 3. Simulation results under 9 dose-toxicity scenarios with the target DLT probability of 0.2. The doses in bold are the MTD. To reflect what may happen in practice, the MTD does not necessarily have the DLT probability equal to the target DLT probability. CRM-nd and BOIN-nd refer to CRM and BOIN with no cohort deviations.

Method	Scenario	Dose Level				
		1	2	3	4	5
	1	**0.2**	0.35	0.4	0.5	0.6
3+3	Selection %	42.7	17.2	5.8	0.8	0.0
	No. of patients	13.2	6.2	2.1	0.5	0.1
CRM-nd	Selection %	54.6	17.3	2.1	0.2	0.0
	No. of patients	16.3	6.2	1.6	0.3	0.0
CRM	Selection %	54.1	16.9	2.4	0.1	0.0
	No. of patients	16.2	6.1	1.5	0.2	0.0
BOIN-nd	Selection %	61.2	14.9	2.7	0.4	0.0
	No. of patients	17.5	5.7	1.4	0.3	0.0
BOIN	Selection %	63.2	14.7	2.2	0.3	0.0
	No. of patients	18.1	5.6	1.2	0.2	0.0
	2	**0.14**	0.32	0.35	0.45	0.55
3+3	Selection %	48.5	19.9	9.9	2.1	0.0
	No. of patients	14.2	7.2	3.0	0.9	0.2
CRM-nd	Selection %	51.0	31.2	6.4	0.6	0.0
	No. of patients	14.9	8.5	2.6	0.6	0.1
CRM	Selection %	52.1	31.8	5.7	0.7	0.0
	No. of patients	15.1	8.7	2.5	0.5	0.1
BOIN-nd	Selection %	58.1	26.9	6.4	0.9	0.1
	No. of patients	16.2	8.1	2.3	0.6	0.1
BOIN	Selection %	61.9	25.0	5.2	0.9	0.1
	No. of patients	16.9	8.0	1.9	0.4	0.0
	3	0.1	**0.2**	0.35	0.45	0.5
3+3	Selection %	29.3	39.3	16.4	2.7	0.0
	No. of patients	9.9	10.5	5.2	1.4	0.2
CRM-nd	Selection %	21.9	54.8	18.1	1.4	0.1
	No. of patients	9.8	11.6	5.5	1.1	0.2
CRM	Selection %	21.5	55.3	17.6	1.8	0.1
	No. of patients	9.9	11.6	5.4	0.9	0.1
BOIN-nd	Selection %	25.9	53.6	15.4	1.8	0.3
	No. of patients	11.2	11.3	4.6	1.0	0.2
BOIN	Selection %	27.9	52.8	14.5	1.8	0.2
	No. of patients	11.8	11.2	4.3	0.8	0.1
	4	0.01	**0.08**	0.35	0.45	0.55
3+3	Selection %	7.7	63.3	22.5	4.9	0.0
	No. of patients	4.9	14.8	7.6	2.2	0.4
CRM-nd	Selection %	0.7	50.4	46.3	2.4	0.2
	No. of patients	3.8	12.0	10.8	2.1	0.4
CRM	Selection %	0.8	51.6	45.0	2.4	0.2
	No. of patients	4.1	11.9	10.7	1.9	0.2

© 2021 Park M et al. *JAMA Network Open.*

BOIN-nd	Selection %	1.9	63.7	31.2	3.0	0.2
	No. of patients	4.4	14.4	8.2	1.7	0.3
BOIN	Selection %	1.5	63.5	31.8	2.9	0.2
	No. of patients	4.8	14.3	8.1	1.4	0.2
	5	0.04	0.06	**0.2**	0.35	0.48
3+3	Selection %	4.3	30.6	41.8	16.0	0.0
	No. of patients	4.5	8.8	9.8	4.8	1.1
CRM-nd	Selection %	0.4	16.0	63.1	18.5	1.6
	No. of patients	4.0	7.0	11.7	5.0	1.1
CRM	Selection %	0.3	17.1	61.8	18.7	1.7
	No. of patients	4.4	7.1	11.3	4.8	1.0
BOIN-nd	Selection %	1.3	23.5	55.9	17.3	1.7
	No. of patients	4.5	8.7	10.3	4.4	1.0
BOIN	Selection %	1.0	23.5	56.8	16.7	1.5
	No. of patients	5.0	9.1	10.1	3.8	0.7
	6	0.01	0.07	**0.12**	0.37	0.5
3+3	Selection %	5.6	13.2	57.1	18.3	0.0
	No. of patients	4.5	6.0	11.6	6.0	1.3
CRM-nd	Selection %	0.1	6.4	60.7	31.4	1.4
	No. of patients	3.4	5.3	12.0	7.1	1.4
CRM	Selection %	0.0	6.5	60.4	31.5	1.7
	No. of patients	3.7	5.7	11.2	6.9	1.2
BOIN-nd	Selection %	1.5	9.5	63.1	24.5	1.4
	No. of patients	4.1	6.4	11.5	5.9	1.1
BOIN	Selection %	1.1	10.1	62.4	24.8	1.6
	No. of patients	4.5	7.0	11.0	5.4	0.9
	7	0.05	0.06	0.09	**0.2**	0.37
3+3	Selection %	4.0	7.9	27.8	34.6	0.0
	No. of patients	4.6	5.0	7.3	7.4	2.8
CRM-nd	Selection %	0.3	4.7	27.9	50.7	15.7
	No. of patients	4.2	4.7	7.5	8.3	4.2
CRM	Selection %	0.4	5.4	28.5	49.4	15.7
	No. of patients	4.7	5.2	7.4	7.6	3.6
BOIN-nd	Selection %	1.6	5.2	27.4	51.5	13.7
	No. of patients	4.7	5.2	7.6	7.8	3.5
BOIN	Selection %	1.3	6.3	29.4	48.3	14.1
	No. of patients	5.3	5.8	7.8	7.0	2.8
	8	0.01	0.02	0.04	**0.08**	0.38
3+3	Selection %	0.6	2.3	6.7	59.1	0.0
	No. of patients	3.5	3.9	4.5	10.3	4.3
CRM-nd	Selection %	0.0	0.1	3.3	56.3	40.3
	No. of patients	3.0	3.2	4.3	9.9	8.6
CRM	Selection %	0.0	0.1	4.1	56.0	39.8
	No. of patients	3.5	3.7	4.6	8.9	8.0
BOIN-nd	Selection %	0.1	0.5	4.3	67.5	27.6
	No. of patients	3.1	3.6	4.7	11.0	6.5

© 2021 Park M et al. *JAMA Network Open.*

BOIN	Selection %	0.0	0.5	4.7	64.8	30.0
	No. of patients	3.7	4.1	5.2	10.0	5.9
	9	0.05	0.06	0.07	0.08	**0.2**
3+3	Selection %	3.9	4.6	5.4	23.8	0.0
	No. of patients	4.5	4.5	4.3	5.8	3.8
CRM-nd	Selection %	0.3	3.8	10.4	25.1	59.6
	No. of patients	4.2	4.5	5.2	5.8	9.2
CRM	Selection %	0.4	4.8	11.1	26.2	56.8
	No. of patients	4.7	5.1	5.5	5.5	7.8
BOIN-nd	Selection %	1.6	3.7	8.0	27.5	58.6
	No. of patients	4.7	4.8	4.8	6.3	8.3
BOIN	Selection %	1.3	4.9	9.6	29.6	54.1
	No. of patients	5.3	5.4	5.2	6.1	6.7

© 2021 Park M et al. *JAMA Network Open.*

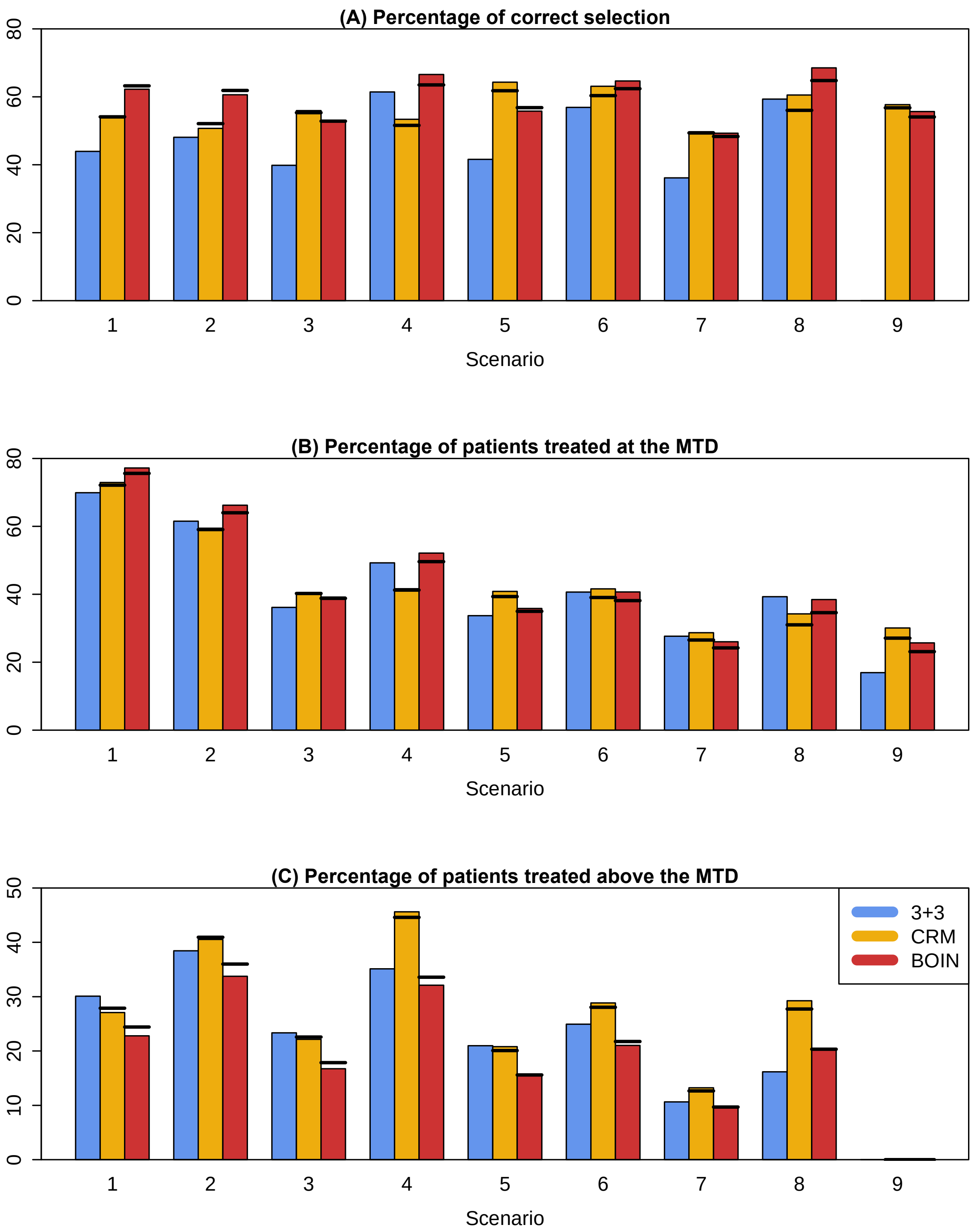

eFigure 2. Simulation results of the 3+3, continual reassessment method (CRM), and bayesian optimal interval (BOIN) designs when the cohort deviation was generated according to the frequency observed in real trials. The bars show the performance (percentage) of the designs when the planned cohort size was strictly followed, and horizontal lines indicate the value of the performance metric in the presence of cohort size deviation. The target DLT rate was 0.2. MTD indicates maximum tolerated dose.

© 2021 Park M et al. *JAMA Network Open.*

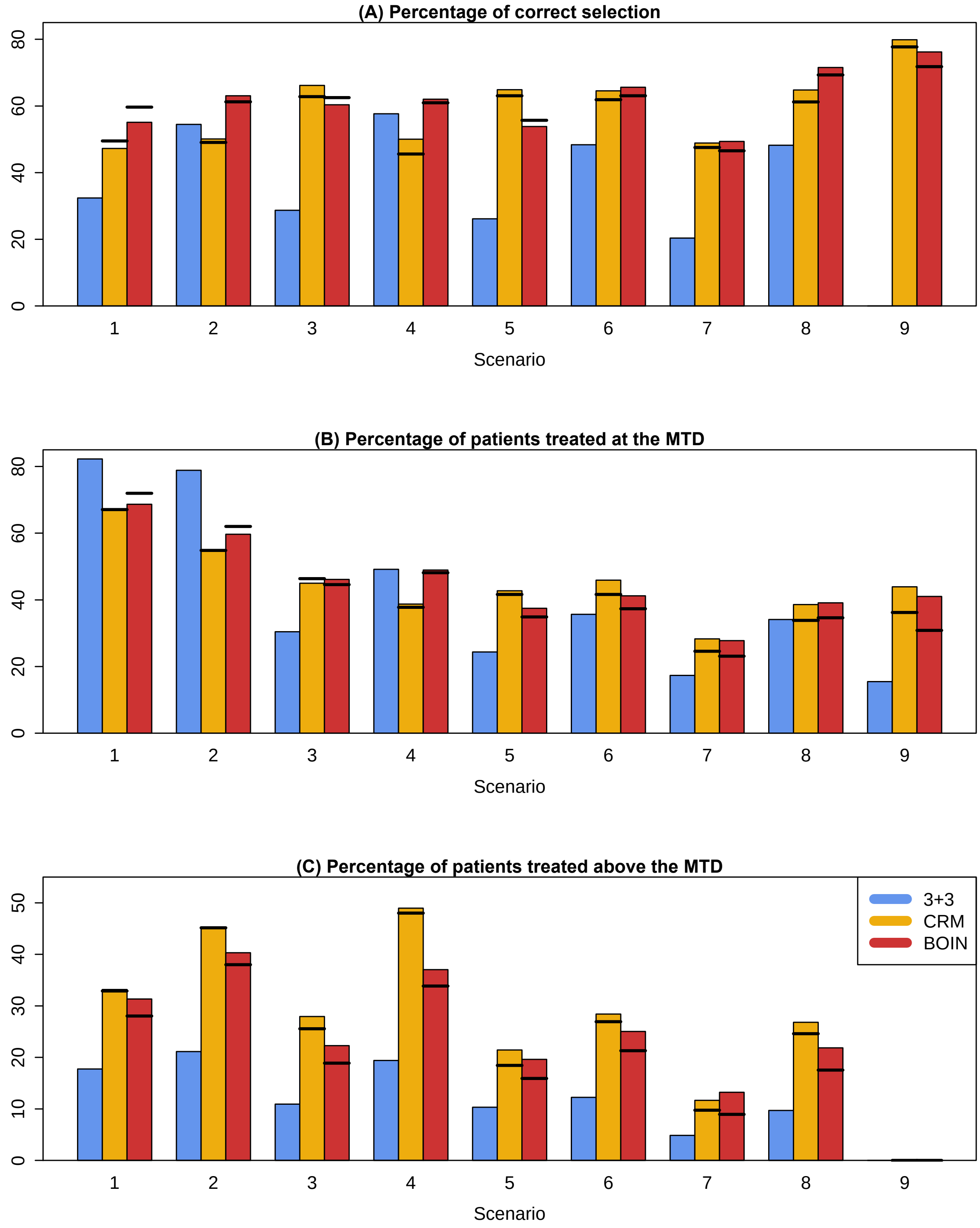

eFigure 3. Sensitivity analysis when the percentage of cohort deviation is increased from 25.7% (the rate observed in the literature review) to 50%, with the target DLT rate of 0.3. The bars show the performance of the designs when the planned cohort size was strictly followed, and horizontal lines indicate the value of the performance metric in the presence of cohort size deviation. MTD indicates maximum tolerated dose.

© 2021 Park M et al. *JAMA Network Open.*

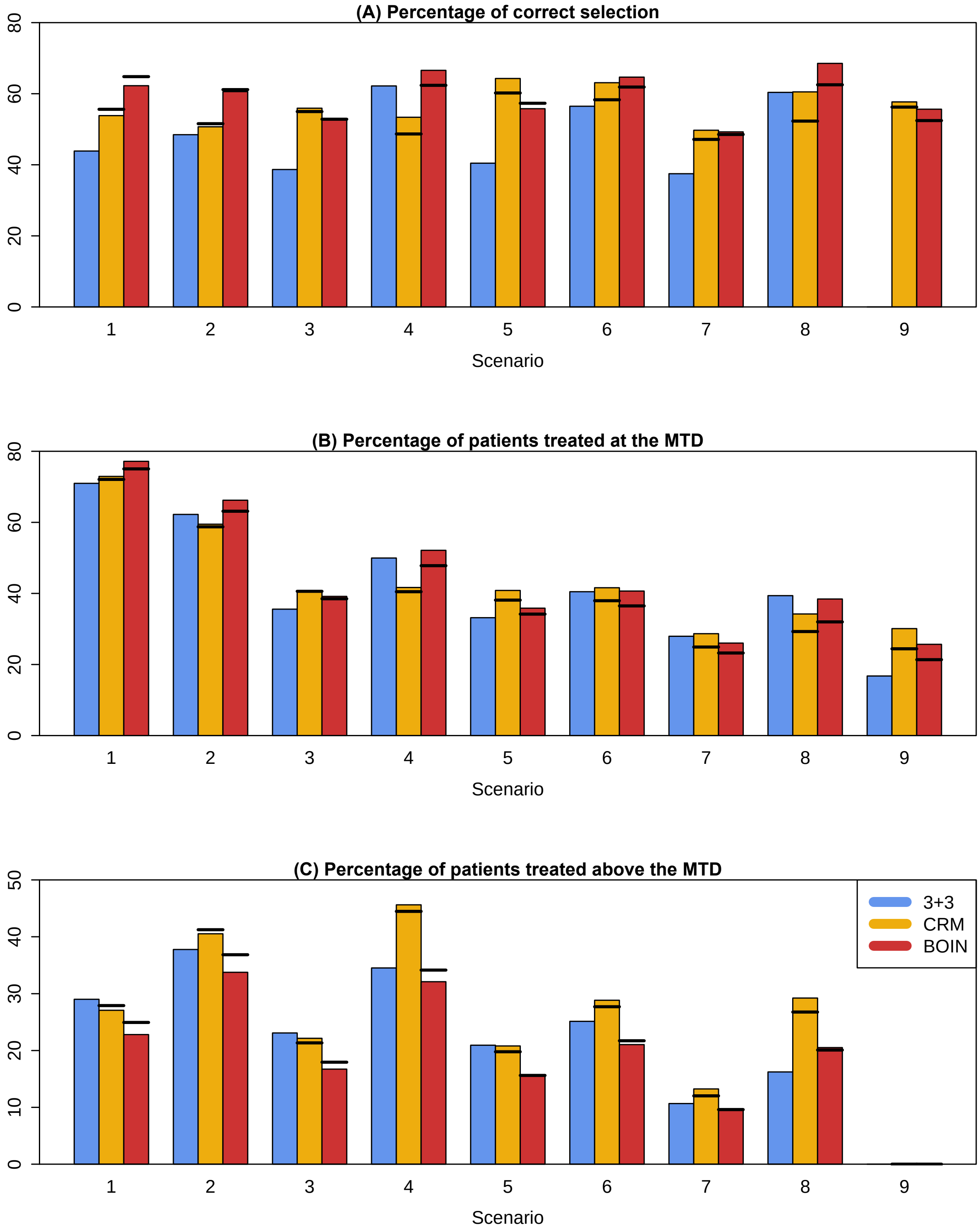

eFigure 4. Sensitivity analysis when the percentage of cohort deviation was increased from 25.7% (the rate observed in the literature review) to 50%, with the target DLT rate of 0.2. The bars show the performance of the designs when the planned cohort size was strictly followed, and the horizontal lines indicate the value of the performance metric in the presence of cohort size deviation. MTD indicates maximum tolerated dose.

© 2021 Park M et al. *JAMA Network Open.*

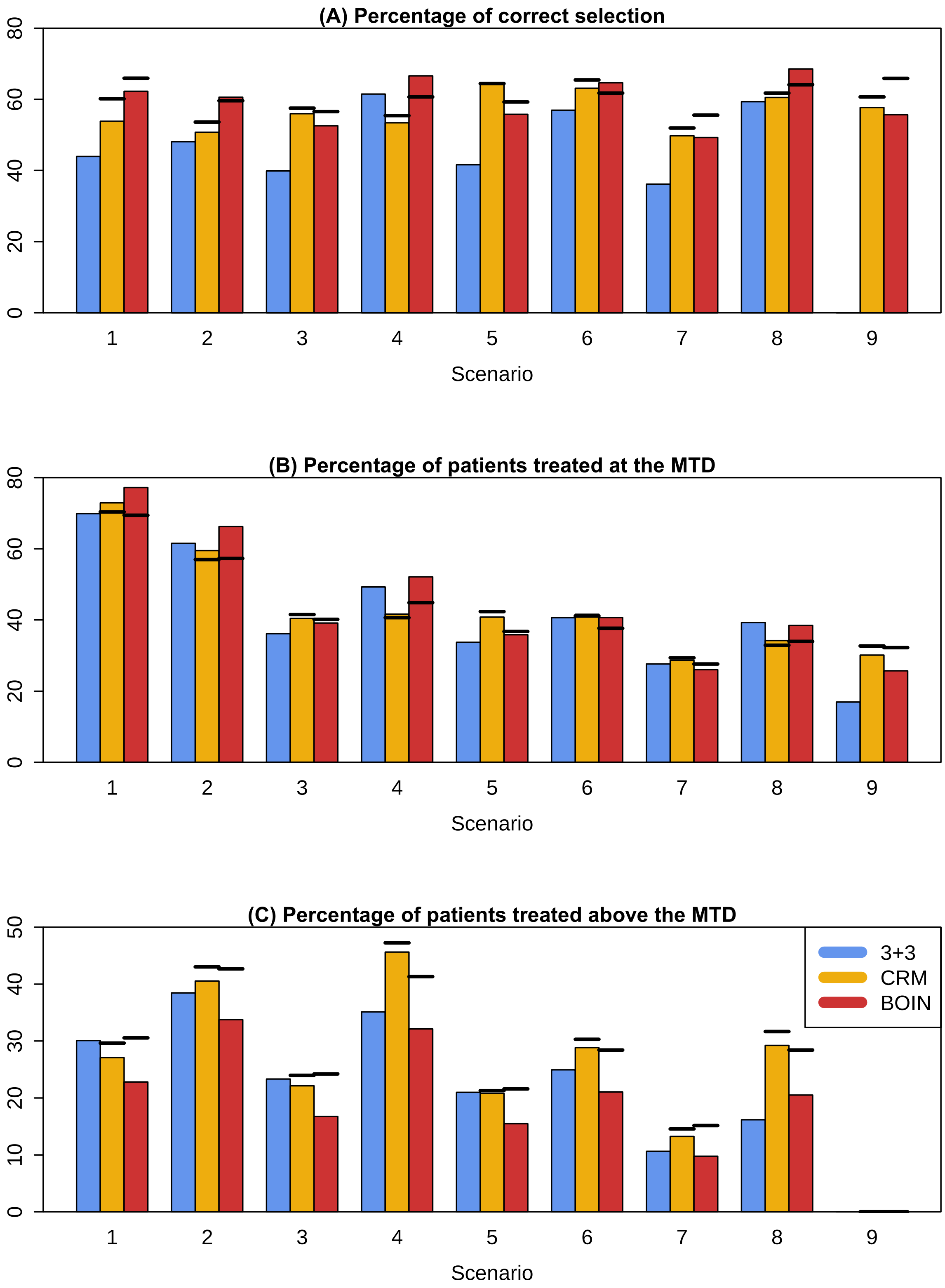

eFigure 5. Simulation results of the 3+3, continual reassessment method (CRM), and bayesian optimal interval (BOIN) designs under informative cohort size deviation with expansion of the next cohort size. If any DLT was observed in the current cohort, the size of the next cohort was expanded to 4. The bars show the performance of the designs when the planned cohort size was strictly followed, and horizontal lines indicate the value of the performance metric in the presence of cohort size deviation. The target DLT rate was 0.2. MTD indicates maximum tolerated dose.

© 2021 Park M et al. *JAMA Network Open.*

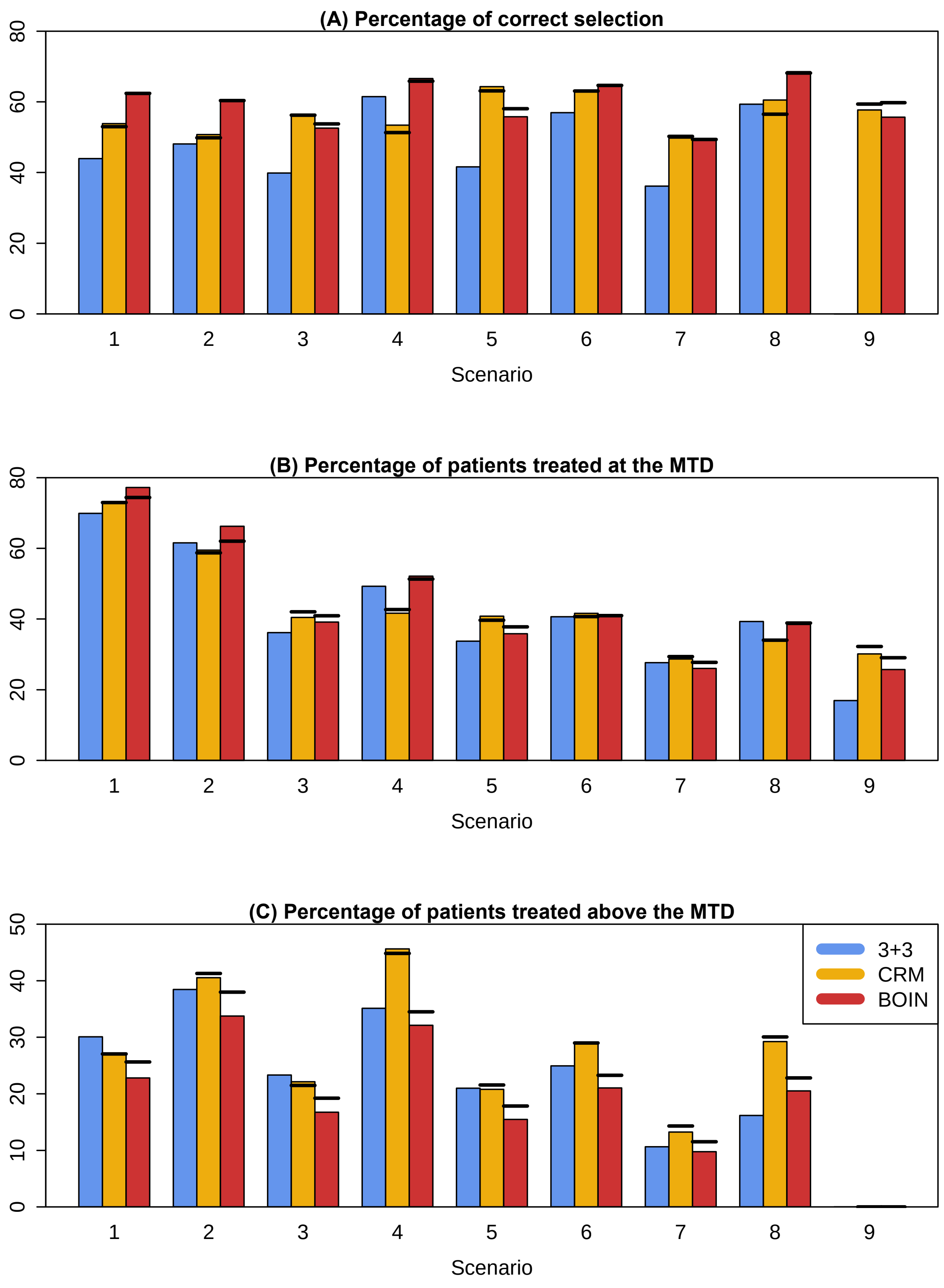

eFigure 6. Simulation results of the 3+3, continual reassessment method (CRM), and bayesian optimal interval (BOIN) designs under informative cohort size deviation with reduction of the next cohort size. If any DLT was observed in the current cohort, the size of the next cohort was reduced to 2. The bars show the performance of the designs when the planned cohort size was strictly followed, and horizontal lines indicate the value of the performance metric in the presence of cohort size deviation. The target DLT rate was 0.2. MTD indicates maximum tolerated dose.

© 2021 Park M et al. *JAMA Network Open.*

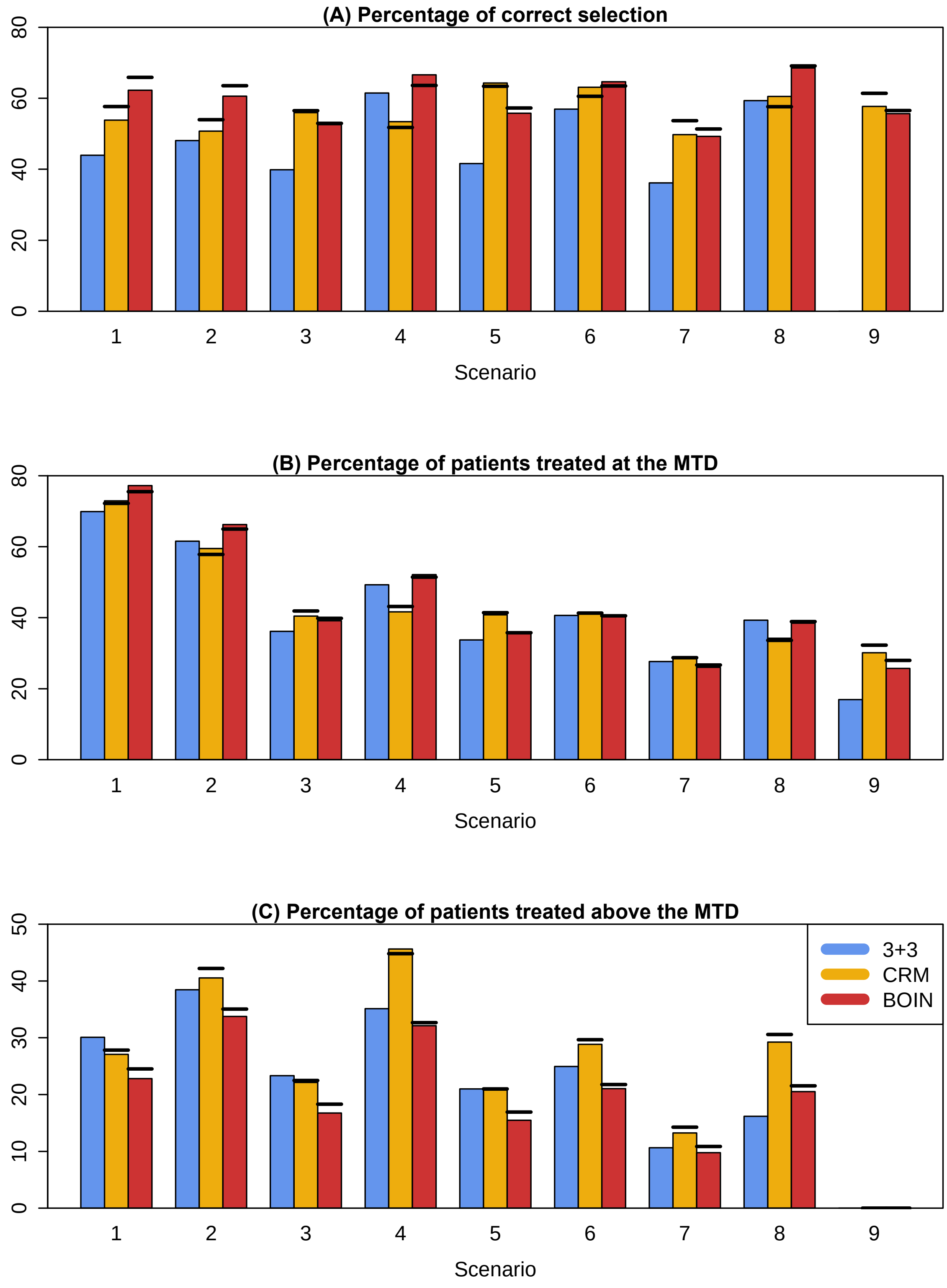

eFigure 7. Simulation results of the 3+3, continual reassessment method (CRM), and bayesian optimal interval (BOIN) designs under informative cohort size deviation with expansion of the present cohort size. If any DLT was observed in the current cohort, the size of the current cohort was expanded to 4. The bars show the performance of the designs when the planned cohort size was strictly followed, and horizontal lines indicate the value of the performance metric in the presence of cohort size deviation. The target DLT rate was 0.2. MTD indicates maximum tolerated dose.

© 2021 Park M et al. *JAMA Network Open.*
